# Sex composition as an unaddressed confound in FDG-PET predictive models of Alzheimer's disease conversion

**DOI:** 10.1016/j.jnha.2026.100955

**Published:** 2026-08-14

**Authors:** Thorsten Rudroff

**Affiliations:** Turku PET Centre, University of Turku and Turku University Hospital, Turku, Finland

Dear Editor,

Alhasan and colleagues report an impressive predictive framework using baseline FDG-PET to forecast cognitive decline and Alzheimer's disease conversion in 4,732 ADNI participants [1]. The variance partitioning rigor of this work, including subject-grouped cross-validation, calibration assessment, and sensitivity analyses, represents a methodological standard the field should emulate. I write to highlight a validity threat embedded in the cohort itself that the predictive models do not address sex composition varies systematically across the diagnostic groups underlying these models, and sex is never tested as an effect modifier of the central FDG-decline relationship.

The authors' own Table 1 reports that female representation falls from 52.3% in cognitively normal participants to 41.9% in MCI and 45.7% in Alzheimer's disease groups [1]. This is not a minor demographic note. It means the reference distribution against which FDG MetaROI z-scores are calculated, and the diagnostic-group comparisons driving the headline four-fold conversion risk finding, are built on a cohort whose sex composition shifts with disease status. If biological sex independently modulates baseline regional glucose metabolism, as multiple independent FDG-PET cohorts have demonstrated, then a model that does not test sex as a covariate or effect modifier cannot distinguish disease-driven hypometabolism from sex-driven differences in the underlying MetaROI distribution.

This is not a hypothetical concern. A recently published variance partitioning analysis of FDG-PET data found that biological sex explained 29.71% of regional glucose metabolic variance in the globus pallidus, while the primary clinical grouping variable explained effectively 0% [2]. Across conditions including multiple sclerosis and Long COVID, biological sex accounts for approximately 30 times more metabolic variance in frontal-striatal circuits than diagnostic category itself [3]. These findings are directly relevant to Alzheimer's disease: sex differences in amyloid burden, tau deposition, and metabolic decline trajectories are documented at the biomarker level, and women with equivalent tau burden show greater metabolic decline than men with comparable biomarker levels [4]. The WYHU problem, the systematic over-reliance on Western, young, healthy, and university-affiliated samples as normative references, and the resulting erasure of individual neurobiological heterogeneity through group-level analysis have been identified as structurally compounding validity threats in Alzheimer's disease biomarker research specifically [5]. The ADNI cohort, as the authors acknowledge, is approximately 89% non-Hispanic White, highly educated, and academically recruited, a demographic profile that compounds the sex composition issue identified here.

Given that women constitute roughly two thirds of Alzheimer's disease cases overall yet are underrepresented in the MCI and Alzheimer's disease groups of this specific cohort relative to the cognitively normal group, the possibility that sex composition differences across diagnostic strata partially drive the reported AUC of 0.826 and the four-fold conversion risk ratio cannot be excluded without a sex-stratified or sex-adjusted reanalysis. Given the size and statistical sophistication of this cohort, I would encourage the authors to report whether sex was tested as a covariate or interaction term in the mixed-effects and predictive models, and if not, to consider a sex-stratified reanalysis. ADNI's open data structure makes this directly feasible and would substantially strengthen the clinical translatability the authors rightly emphasize.

## CRediT authorship contribution statement

Thorsten Rudroff: Conceptualization, Writing – original draft, Writing – review & editing.

## Declaration of Generative AI and AI-assisted technologies in the writing process

Claude (Anthropic) was used for language editing only. All scientific content, analysis, and conclusions are solely those of the author.

## Declaration of competing interest

The author declares that he has no known competing financial interests or personal relationships that could have appeared to influence the work reported in this paper.
